# Inhibition of tRNA Gene Transcription by the Immunosuppressant Mycophenolic Acid

**DOI:** 10.1128/MCB.00294-19

**Published:** 2019-12-11

**Authors:** Aneta Jurkiewicz, Ewa Leśniewska, Małgorzata Cieśla, Neuton Gorjão, Theodoros Kantidakis, Robert J. White, Magdalena Boguta, Damian Graczyk

**Affiliations:** a Institute of Biochemistry and Biophysics, Polish Academy of Sciences, Warsaw, Poland; b Aston Medical Research Institute, Aston Medical School, Aston University, Birmingham, United Kingdom; c Department of Biology, University of York, York, United Kingdom

**Keywords:** RNA polymerase III, tRNA, mycophenolic acid, yeast, macrophages

## Abstract

Mycophenolic acid (MPA) is the active metabolite of mycophenolate mofetil, a drug that is widely used for immunosuppression in organ transplantation and autoimmune diseases, as well as anticancer chemotherapy. It inhibits IMP dehydrogenase, a rate-limiting enzyme in *de novo* synthesis of guanidine nucleotides. MPA treatment interferes with transcription elongation, resulting in a drastic reduction of pre-rRNA and pre-tRNA synthesis, the disruption of the nucleolus, and consequently cell cycle arrest. Here, we investigated the mechanism whereby MPA inhibits RNA polymerase III (Pol III) activity, in both yeast and mammalian cells. We show that MPA rapidly inhibits Pol III by depleting GTP. Although MPA treatment can activate p53, this is not required for Pol III transcriptional inhibition. The Pol III repressor MAF1 is also not responsible for inhibiting Pol III in response to MPA treatment. We show that upon MPA treatment, the levels of selected Pol III subunits decrease, but this is secondary to transcriptional inhibition. Chromatin immunoprecipitation (ChIP) experiments show that Pol III does not fully dissociate from tRNA genes in yeast treated with MPA, even though there is a sharp decrease in the levels of newly transcribed tRNAs. We propose that in yeast, GTP depletion may lead to Pol III stalling.

## Abstract (precis)

Mycophenolic acid (MPA) is the active metabolite of mycophenolate mofetil, a drug that is widely used for immunosuppression in organ transplantation and autoimmune diseases, as well as anticancer chemotherapy. It inhibits IMP dehydrogenase, a rate-limiting enzyme in *de novo* synthesis of guanidine nucleotides.

## INTRODUCTION

Mycophenolate mofetil (MMF) is a highly effective immunosuppressive prodrug that is used widely and in a range of clinical contexts, including organ transplantation, autoimmune disease, and cancer therapy (1, 2). Its active metabolite, mycophenolic acid (MPA), inhibits IMP dehydrogenase (IMPDH). IMPDH catalyzes the NAD-dependent oxidation of IMP to XMP, which is a rate-limiting step in the *de novo* guanosine nucleotide synthesis pathway. This pathway utilizes glucose and amino acids to generate GTP (2). The clinical relevance of MPA is based on the fact that inhibition of IMPDH impacts especially on B and T lymphocytes, which depend singularly on the *de novo* pathway for purine synthesis, instead of using the salvage pathway (3). T and B lymphocytes play a key role in acute and chronic antigen-dependent transplant rejection (4). It has now become clear, however, that myeloid cells such as monocytes, dendritic cells, and macrophages also play an important role in this process (4, 5).

In the yeast Saccharomyces cerevisiae, there are four paralogous genes encoding IMP dehydrogenases (*IMD1* to *IMD4*). Because *IMD1* is very close to the telomere, and it contains a frameshift insertion, it is considered to be a pseudogene (6). *IMD2* and, to a lesser extent, *IMD4* are induced in the presence of guanidine nucleotide-depleting drugs. Interestingly, when overexpressed, only *IMD2* confers resistance to these drugs (6, 7). In humans and other mammals, two isoforms of the *IMPDH* gene exist, *IMPDH1* and *IMPDH2*. The products of these genes are kinetically indistinguishable and are highly similar, being 84% identical at the protein level in humans. Whereas *IMPDH1* is constitutively expressed at low levels in virtually all tissues, *IMPDH2* is inducible and generally expressed in highly proliferative cells (8).

IMPDH inhibitors 6-azauracil (6-AU) and MPA reduce GTP levels and in doing so lead to transcription elongation defects by limiting a transcription substrate (9). Transcription in eukaryotic cells is directed by at least three different multimeric RNA polymerases (Pols). Pol I is responsible for synthesis of rRNA. Pol II transcribes mRNAs and also most small nuclear RNAs (snRNAs) and microRNAs (miRNAs). Pol III synthesizes tRNA, 5S rRNA, 7SL RNA, and a subset of small noncoding RNAs required for the maturation of other RNA molecules (e.g., U6 snRNA). Nucleotide depletion differentially impacts the three RNA polymerases and their RNA product levels. Treatment of yeast cells by 6-AU leads to the rapid cessation of Pol I and Pol III activity, whereas Pol II seems to be less affected, probably owing to the lower rate of transcription (10). In mammalian cells, GTP depletion by MPA also specifically leads to Pol I and Pol III inhibition (11). Therefore, nucleotide depletion leads to imbalances between precursors of mRNA, rRNA, and tRNA. The consequence of nucleotide depletion, in both yeast and mammalian cells, is a nucleolar stress and cell cycle arrest. In mammalian cells, the cell cycle arrest is induced by p53, which is activated as a result of free L5 and L11 ribosomal proteins binding to Mdm2 E3 ubiquitin ligase, which normally targets p53 for degradation (11).

Pol III in yeast is negatively regulated by a general repressor, Maf1 (12). Maf1 integrates multiple signaling pathways and inhibits Pol III in response to nutrient limitation or stress conditions. Interestingly, in yeast, all so-far-tested stress conditions that repress Pol III activity do so through Maf1 (13, 14). Maf1 is also conserved in higher eukaryotes, where it plays a similar role in regard to Pol III (for review, see reference 14 and references therein). However, in these organisms, Pol III is also directly inhibited by p53 and RB and activated by c-Myc, mTORC, and extracellular signal-regulated kinase (ERK) (15–18). Moreover, Pol III transcription has been shown to be directly activated by NF-κB, a key transcription factor mediating inflammatory signals (19). It is, however, unknown whether inhibition of Pol III activity by MPA is an effect of one or more signaling pathways that impinge on Pol III.

Here, we confirm previous observations that MPA inhibits Pol III activity in mammalian cells and show that it also occurs in yeast. We further explore this mechanistically by assaying Pol III association with tRNA genes. We show that in mammalian cells, both tRNA levels and Pol III binding to tRNA genes rapidly decrease upon MPA treatment. Strikingly, in yeast, the rapid decrease of tRNA levels is not fully followed by a dissociation of Pol III from its templates, which may be a result of Pol III stalling. Furthermore, the observed downregulation of Pol III subunit levels and p53 induction in a mouse macrophage cell line are also irrelevant to a drop in tRNA transcription. Finally, we show that the decrease of Pol III activity upon MPA treatment does not depend on Maf1, in either yeast or mammalian cells. Notably, to our best knowledge, this is a first report showing that a stress factor does not involve Maf1 to repress Pol III transcription in yeast.

## RESULTS

### MPA induces rapid inhibition of Pol III activity in eukaryotic cells.

**FIG 1:**
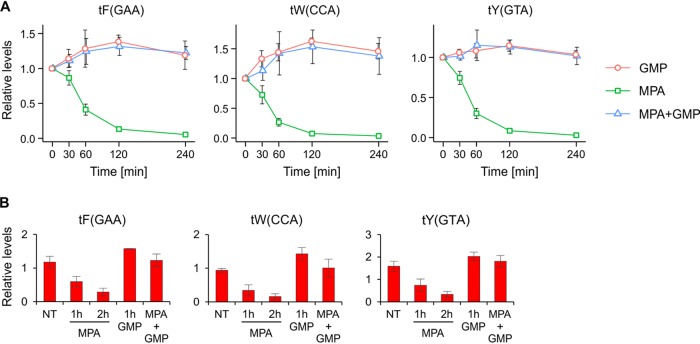
MPA decreases tRNA levels in a macrophage cell line through interfering with the GMP nucleotide synthesis pathway. (A) The RAW 264.7 murine macrophage cell line was treated with either 10 μM MPA, 50 μM GMP, or both for the indicated period. (B) Similarly to those in panel A, RAW 264.7 cells were treated with either 10 μM MPA or 50 μM GMP alone for the indicated period, or cells were first treated with MPA for 1 h and then GMP was added for another hour (MPA + GMP sample). Total RNA was isolated and reverse transcribed, and tRNA levels were measured using qRT-PCR. All samples were normalized to the geometric mean for the ARPP, GAPDH, and ACTB mRNAs. *n* = 3. The error bars represent the standard deviation. NT, not treated.

MPA inhibits Pol I and Pol III transcription *in vivo*, leading to p53-dependent cell cycle arrest in cancer cells (11). Given the relevance of macrophages in transplant rejection, we tested whether MPA affects Pol III activity also in this type of cells. Pol III activity was followed by analyzing the levels of primary transcripts of selected tRNA genes by RT-qPCR. Due to lack of modifications, the primary tRNA transcripts are preferential substrates for reverse transcriptase. As shown in Fig. 1A, MPA rapidly decreases the expression of tRNA in the RAW 264.7 macrophage cell line. To test if the observed effect of MPA on tRNA synthesis results from IMPDH inhibition and is not an effect on another cellular component(s), RAW 264.7 cells were additionally treated with either GMP or both MPA and GMP. Whereas GMP has no effect or modestly increases tRNA levels by itself, it clearly prevents the decrease in tRNA levels upon MPA treatment (Fig. 1A). Moreover, addition of GMP after MPA treatment fully rescued the tRNA levels (Fig. 1B).

**FIG 2:**
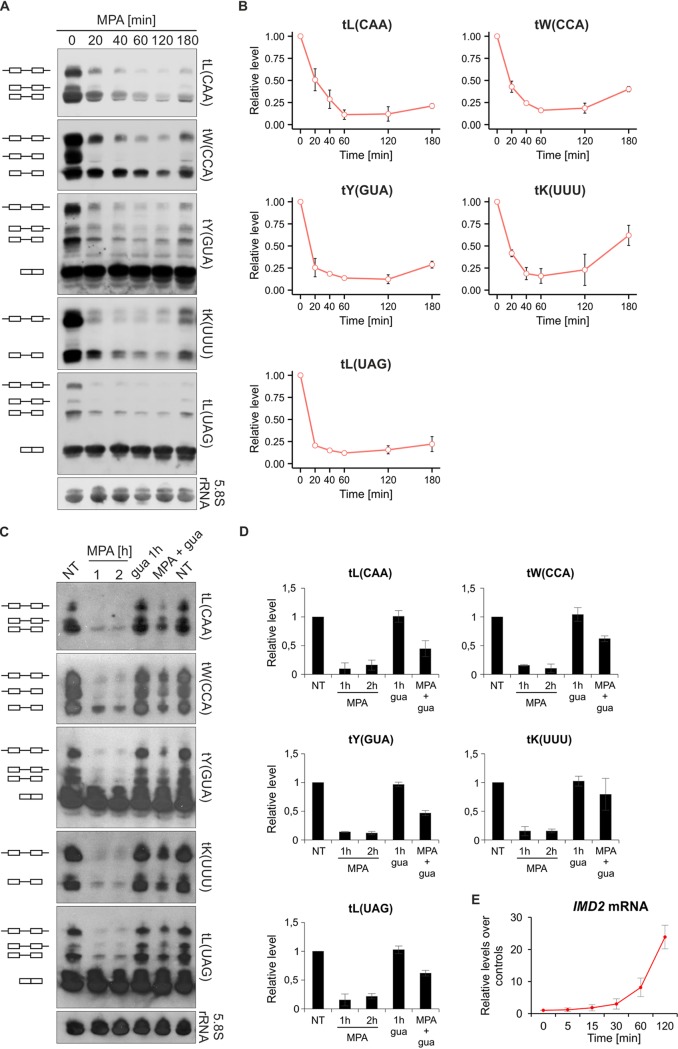
MPA inhibits tRNA synthesis in yeast cells. (A) Rapid decrease of tRNA synthesis by MPA is reactivated upon prolonged treatment. Wild-type yeast cells were grown in minimal medium without uracil to exponential growth phase at 30°C and then treated with 50 μg/ml MPA for the indicated period. RNA was extracted from cells and subjected to Northern blotting. The blot was hybridized with the following probes: tL(CAA), tW(CCA), tY(GUA), tK(UUU), and tL(UAG). 5.8S rRNA was used as a loading control. Pictograms next to the Northern blots represent the following forms of tRNAs: unprocessed primary transcripts, 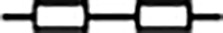; 5′-end processed pre-tRNA, 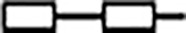; 3′-end processed pre-tRNA, 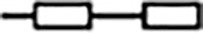; end-matured intron-containing pre-tRNAs, 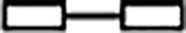; mature tRNA, 
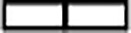
. (B) Kinetics of tRNA synthesis inhibition by MPA. Quantification of pre-tRNA from Northern blots from panel A. *n* = 3. (C) Guanine rescues the low levels of tRNA in MPA-treated yeast cells. Wild-type yeast cells were grown in minimal medium without uracil to exponential growth phase at 30°C and then left untreated (NT) or treated with 100 μg/ml MPA or 0.3 mM guanine (gua) for the indicated period. MPA-treated cell cultures were split in two, and after 1 h, 0.3 mM guanine was added to one sample for another hour (MPA + gua). RNA was extracted from cells and subjected to Northern blotting as in panel A. (D) Quantification of pre-tRNA from Northern blots from panel C. *n* = 2. (E) Treatment of yeast with MPA results in *IMD2* mRNA induction. Wild-type yeast cells were grown in minimal medium without uracil to exponential growth phase at 30°C and then treated with 100 μg/ml MPA for the indicated period. RNA was extracted from cells, and *IMD2* mRNA levels were assessed by RT-qPCR. All samples are normalized to the geometric mean for the ACT1, ALG9, and TDH1 mRNAs. *n* = 3. The error bars represent the standard deviation.

**FIG 3:**
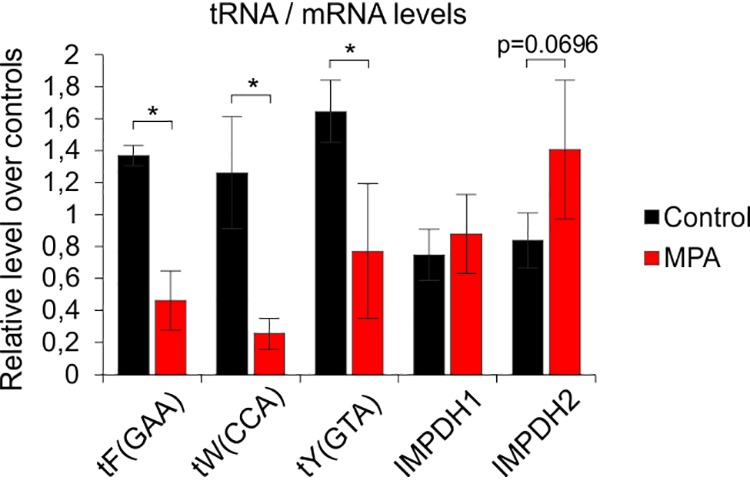
Effects of prolonged MPA treatment on RAW 264.7 macrophage cell line. The RAW 264.7 murine macrophage cell line was treated with either ethyl alcohol (MPA vehicle) or 10 μM MPA for 8 h. Total RNA was isolated, and indicated tRNA and mRNA levels were measured using qRT-PCR. All samples are normalized to the geometric mean for the ARPP, GAPDH, and ACTB mRNAs. *n* = 3. Error bars represent the standard deviation. Asterisk indicates *P* value of <0.05.

Given that nucleotide depletion by 6-AU inhibits Pol III activity (10), we also tested the effect of MPA on tRNA synthesis in yeast. MPA triggers a rapid decrease of tRNA levels, and within 60 min of treatment, the pre-tRNA species are almost undetectable by Northern blotting (Fig. 2A and B). It has been shown that the inhibitory effect of MPA on the cell cycle in yeast can be reversed by addition to the culture medium of guanine, which is converted to GMP by Hpt1 enzyme (20). Consistent with this, we observed rescue of tRNA synthesis in MPA-treated cells after addition of guanine to the culture media (Fig. 2C and D). After 3 h treatment of yeast cells with MPA, partial recovery of tRNA transcription was observed (Fig. 2A and B), which correlated with the induction of the *IMD2* mRNA (Fig. 2E). Possibly, the elevated IMPDH restored the levels of cellular GTP, leading to resumption of Pol III activity even in the presence of MPA in the culture medium. In contrast, in RAW 264.7 macrophages tRNA levels remain very low upon prolonged, 8-h, MPA treatment with no accompanying change in IMPDH1 and only a slight, statistically insignificant, increase in IMPDH2 mRNA levels (Fig. 3). Such a prolonged treatment leads to cell death (see below).

**FIG 4:**
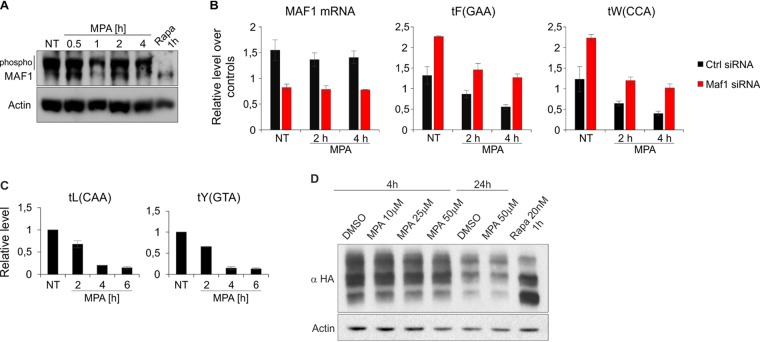
Maf1 is not involved in Pol III inhibition upon MPA treatment in macrophage and tumor cell lines. (A) RAW 264.7 cells transduced with vector expressing the His-Flag-tagged version of MAF1 were left untreated (NT) or treated with 10 μM MPA for the indicated period or 100 nM rapamycin for 1 h. Protein extracts were prepared and resolved on a Phos-tagged SDS-PAGE gel to visualize phosphorylated forms of MAF1. (B) The RAW 264.7 murine macrophage cell line was transfected with either nontargeting siRNA or an siRNA pool targeting Maf1. The cells were then left untreated or treated with 10 μM MPA for the indicated period. Total RNA was isolated and reverse transcribed, and Maf1 mRNA and the levels of indicated tRNAs were measured using qRT-PCR. All samples were normalized to the geometric mean for the ARPP, GAPDH, and ACTB mRNAs. *n* = 3. The error bars represent the standard deviation. (C) U2OS human osteosarcoma cells were either left untreated (NT) or treated with 10 μM MPA for the indicated period. Total RNA was isolated, and tRNA levels were measured using qRT-PCR. All samples are normalized to the geometric mean of the ARPP and ACTB mRNAs and 18S rRNA. *n* = 2. The error bars represent the standard deviation. (D) U2OS cells transfected with an HA-tagged version of MAF1 were treated with either dimethyl sulfoxide (DMSO) or the indicated concentrations of MPA for the indicated period or 20 nM rapamycin for 1 h. Protein extracts were prepared and resolved on a Phos-tagged SDS-PAGE gel as in panel A.

Overall, these data suggest that MPA induces very rapid cessation of Pol III activity in both yeast and mammalian cells (see also Fig. 4C and Fig. 8B), and this effect is most likely the result of inhibition of GMP synthesis.

### Maf1 is not involved in tRNA regulation upon MPA treatment.

Maf1 is a negative regulator of Pol III in all eukaryotic cells investigated (14). Maf1 itself is regulated by phosphorylation, and its dephosphorylated form is known to inhibit Pol III (14). To study the role of MAF1 in the decrease of tRNA levels upon MPA treatment in the RAW 264.7 macrophage cell line, we first looked at its phosphorylation status by resolution of protein extracts on Phos-tag gels (Fig. 4A). Slower-migrating bands, corresponding to phosphorylated forms of MAF1 that are observed in untreated cells, disappear when cells are treated with rapamycin, which is known to block MAF1 phosphorylation and inhibit Pol III (21–23). In contrast, MPA treatment has no effect on MAF1 phosphorylation status in RAW 264.7 macrophages. Similarly, while MPA efficiently decreased tRNA levels in the human osteosarcoma cell line U2OS (Fig. 4C), it had no effect on MAF1 phosphorylation in these cells (Fig. 4D). It is, however, possible that MPA induces only modest phosphorylation changes to MAF1, which may be imperceptible by Phos-tag gels and still have an effect on Pol III activity. We therefore knocked down MAF1 in RAW 264.7 macrophages using small interfering RNA (siRNA). As shown in Fig. 4B, siRNA treatment resulted in the downregulation of *Maf1* mRNA. Concomitantly, we observed increased tRNA levels, suggesting the effectiveness of MAF1 knockdown. Addition of MPA to the cells led to a decrease in tRNA levels in both the nontargeting and MAF1-targeting siRNA-treated cells (Fig. 4B). Thus, depletion of MAF1 does not prevent inhibition by MPA.

**FIG 5:**
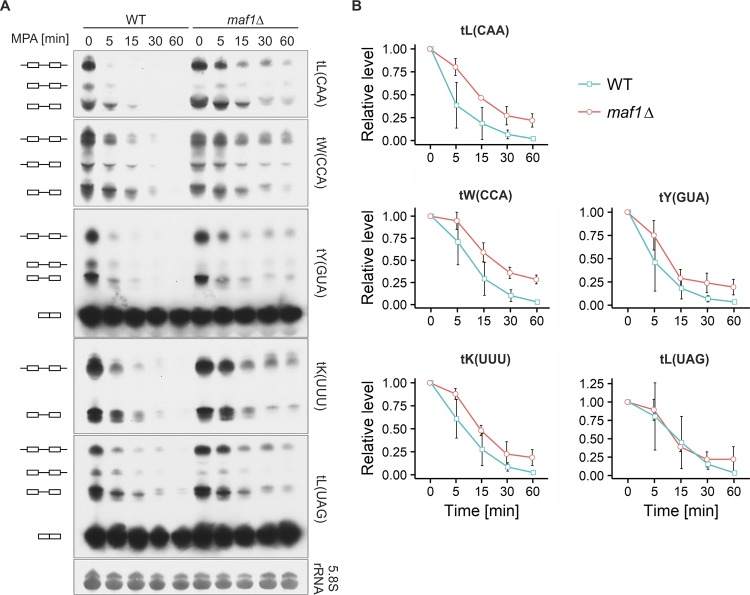
Inhibition of tRNA synthesis by MPA treatment of yeast is Maf1 independent. Wild-type and *maf1Δ* yeast cells were grown in minimal medium without uracil to exponential growth phase at 30°C and then treated with 100 μg/ml MPA for the indicated period. RNA was extracted from cells and subjected to Northern blot analysis. The blot was hybridized with the following probes: tL(CAA), tW(CCA), tY(GUA), tK(UUU), and tL(UAG). 5.8S rRNA was used as a loading control. (A) Representative Northern blots. Pictograms are as defined for Fig. 2A. (B) Quantification of pre-tRNA levels. Pre-tRNA levels were normalized to 5.8S rRNA, and a sample from time zero was set to 1. *n* = 3. Error bars represent the standard deviation.

The potential role of Maf1 in the inhibition of Pol III activity by MPA was also tested using the yeast model. To this end, the kinetics of Pol III inhibition were compared in wild-type and *maf1Δ* mutant cells. Time-course MPA treatment followed by Northern blotting analysis demonstrated fast and strong reduction in the levels of various pre-tRNAs in both wild-type and *maf1Δ* cells (Fig. 5A). The inhibition of tRNA synthesis was a little less prominent in cells with *MAF1* inactivation (Fig. 5B). A drop in pre-tRNA levels can result from two processes, a decrease of pre-tRNA synthesis and their processing into mature tRNAs. It has been shown previously that in the absence of Maf1, the processing of tRNA precursors is delayed (24, 25). Thus, the slightly slower kinetics of the decrease in pre-tRNA levels in yeast *maf1Δ* mutant treated with MPA could be explained by the slower tRNA maturation.

Overall, the results suggest that Maf1 is not required for Pol III inhibition upon MPA treatment either in yeast or in a mouse macrophage cell line.

### Downregulation of Pol III subunit levels as a consequence of Pol III inhibition by MPA.

Inhibition of transcription may lead to Pol III degradation (26). The multistep pathway described for defective yeast Pol III, inactivated by mutations or through decreased expression, involves sumoylation and ubiquitylation of selected subunits. This triggers disassembly of Pol III followed by proteosomal degradation of its largest subunit, C160 (26). Ubiquitylation and proteosomal degradation of C160 were also observed as a consequence of Pol III repression upon physiological stress, which pertains to most of the polymerase complexes that dissociate from templates (27). Surprisingly, the interactions between Pol III subunits in the complexes that were retained under stress conditions became even more tight (27).

**FIG 6:**
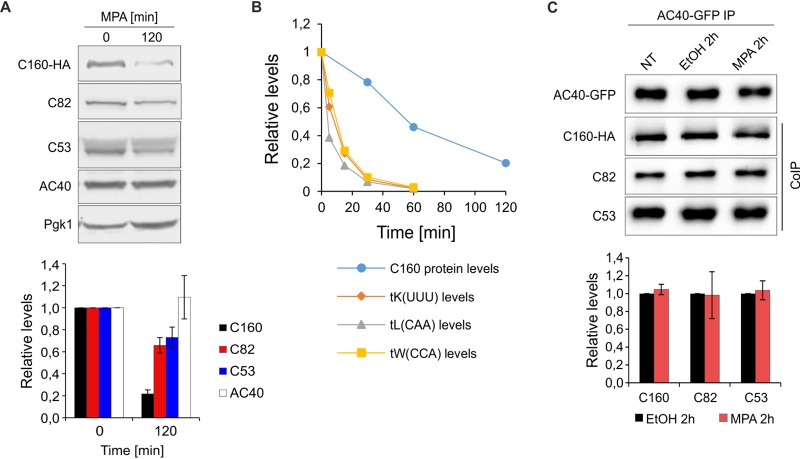
Modulation of Pol III subunit levels as a consequence of Pol III inhibition by MPA. (A) Wild-type yeast cells expressing HA-tagged C160 subunit were grown in minimal medium without uracil to exponential growth phase at 30°C and then treated with 100 μg/ml MPA for 2 h. (Top) Representative Western blot showing the levels of indicated Pol III subunits and Pgk1, which was used as a loading control. (Bottom) Quantification of Pol III subunit levels. The mean subunit expression is shown normalized to the Pgk1 protein, and the time zero sample is set to 1. *n* = 3. Error bars represent the standard deviation. (B) Plot comparing the kinetics of tRNA decrease and C160 level downregulation upon MPA treatment. The data for selected tRNA levels from Fig. 5 were used. The data related to C160 levels were taken from the work of Leśniewska et al. (27) and were obtained from the same strain, MW4415, grown and treated similarly as in panel A. The error bars were omitted for the sake of figure clarity. (C) Wild-type yeast cells expressing HA-tagged C160 and GFP-tagged AC40 subunits were grown in minimal medium without uracil to exponential growth phase at 30°C and then left untreated (NT) or treated with either ethyl alcohol (EtOH) or 100 μg/ml MPA for 2 h. Cellular extracts were incubated with magnetic beads coated with anti-GFP antibody. (Top) After extensive washes, immunoprecipitated proteins were eluted and analyzed by Western blotting using antibodies to GFP, HA tag, C82, and C53. (Bottom) Estimation of binding of C160, C82, and C53 to AC40. The binding of the subunits in MPA-treated cells was calculated relative to their binding in EtOH-treated cells, which was set to 1. *n* = 3. Error bars represent the standard deviation.

Here, we tested the levels and interaction between selected Pol III subunits upon treatment of yeast cells with MPA (Fig. 6). MPA elicited a substantial (∼5-fold) decrease in C160 protein levels. Whether the C160 subunit is directed for degradation by the ubiquitin/proteasome system upon MPA treatment remains to be tested. The levels of C82 and C53 also decreased, but to a lesser extent than C160. The levels of the AC40 subunit seemed to be relatively stable. Thus, MPA treatment affects the levels of Pol III subunits to various extents (Fig. 6A). It is unlikely that the decrease of C160 levels is a causative event in Pol III inhibition upon MPA treatment, as the decrease in pre-tRNA levels precedes C160 degradation (Fig. 6B). The entire Pol III complex was immunopurified using green fluorescent protein (GFP)-specific antibody and GFP-tagged AC40 subunit as bait. The interactions between selected Pol III subunits were examined by coimmunoprecipitation followed by Western blotting. Clearly, MPA treatment does not change the levels of coimmunoprecipitated C160, C82, and C53 subunits compared to the respective controls (Fig. 6C), suggesting that in the retained Pol III complexes the interactions between subunits are preserved.

**FIG 7:**
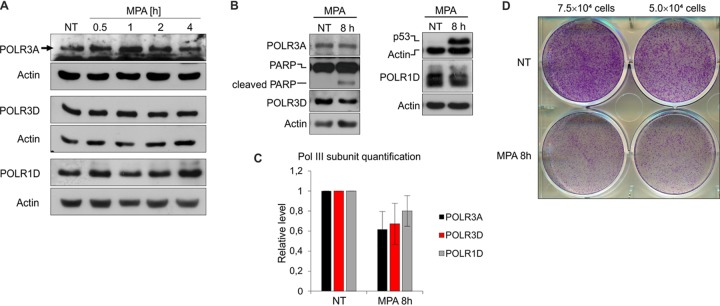
Prolonged MPA treatment induces cell death and modest Pol III subunit downregulation in the RAW 264.7 macrophage cell line. The RAW 264.7 murine macrophage cell line was treated with either ethyl alcohol or 10 μM MPA for up to 4 h (A) or 8 h (B). (A and B) Representative Western blots showing the levels of indicated proteins. The samples were run on an 8 or 15% acrylamide gel. Actin was used as a loading control. (C) Quantification of Pol III subunit levels from panel B. The mean subunit expression is shown normalized to actin, and the time zero sample is set to 1. *n* = 5. Error bars represent the standard deviation. (D) Clonogenic survival assay. Seventy-five thousand or fifty thousand RAW 264.7 cells were plated, allowed to attach overnight, and then treated as for panel B. After treatment, the cells were washed with fresh medium and allowed to grow for another 3 days, followed by staining with crystal violet.

We subsequently tested the levels of selected Pol III subunits in RAW 264.7 macrophages treated with MPA. In contrast to yeast, MPA has no influence on POLR3A, POLR3D, or POLR1D protein levels over 4 h of treatment in these macrophages (Fig. 7A), although transcription by Pol III was inhibited by approximately 90% (Fig. 1). Levels of these Pol III subunits drop slightly, however, after 8-h MPA treatment, which also correlates with the onset of cell death, as manifested by decreased cell survival in clonogenic assays, poly(ADP-ribose) polymerase (PARP) cleavage, and p53 activation (Fig. 7B to D). However, these decreases are too slow to account for the rapid Pol III transcriptional inhibition in RAW 264.7 macrophages under these conditions.

### p53 is not required for the decrease of tRNA levels upon MPA treatment.

p53 is a key tumor suppressor which has been shown to negatively regulate transcription by Pol III in mammalian cells (28). p53 has also been shown to be strongly induced in human cells upon MPA treatment (29).

**FIG 8:**
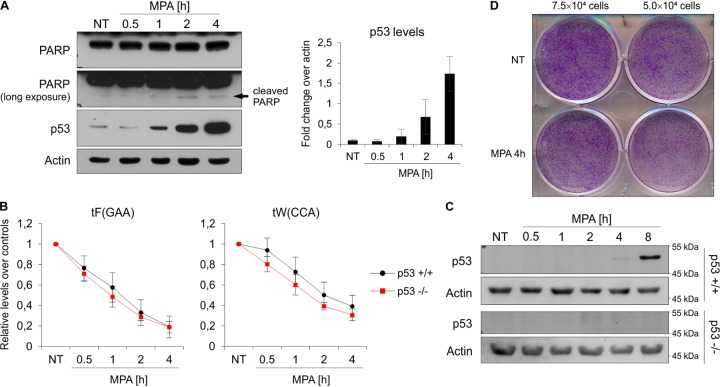
p53 is not required for decrease of tRNA levels upon MPA treatment. (A) The RAW 264.7 murine macrophage cell line was left untreated (NT) or treated with 10 μM MPA for the indicated period. (Left) Representative Western blots showing the levels of indicated proteins. Actin was used as a loading control. (Right) Quantification of p53 levels. The mean p53 expression is shown normalized to actin. *n* = 3. Error bars represent the standard deviation. (B and C) p53-positive (p53 +/+) and p53-null (p53 -/-) HCT116 colorectal cancer cells were treated with 10 μM MPA for the indicated period. (B) Total RNA was isolated, and the levels of indicated mRNAs were measured using qRT-PCR. All samples are normalized to the geometric mean of the ARPP, GAPDH, and ACTB mRNAs, and nontreated (NT) sample is set to 1. *n* = 3. (C) Representative Western blots showing the levels of indicated proteins. Actin was used as a loading control. *n* = 2. (D) Clonogenic survival assay. Seventy-five thousand or fifty thousand RAW 264.7 cells were plated, allowed to attach overnight, and then treated with 10 μM MPA for 4 h. After treatment, the cells were washed with fresh medium and allowed to grow for another 3 days, followed by staining with crystal violet.

Since we saw cell death and p53 activation after 8 h of MPA treatment (Fig. 7), we reasoned that the p53 response may occur more rapidly and contribute to Pol III inhibition in RAW 264.7 macrophages. Indeed, p53 activation occurs much earlier, and its levels begin to rise within 1 h after MPA treatment (Fig. 8A). However, given that we see decreased tRNA levels after only 30 min of MPA treatment (Fig. 1), it is unlikely that p53 is responsible for Pol III inhibition under these conditions. This is further supported by two lines of evidence. First, in the colorectal cancer cell line HCT116 lacking p53, MPA treatment induces similarly rapid tRNA transcription inhibition as in p53-positive HCT116 cells (Fig. 8B). Second, in p53-positive HCT116 cells, p53 upregulation is not apparent within 4 h of MPA treatment and a substantial p53 induction was observed only after 8 h of MPA treatment (Fig. 8C). As expected, the p53 induction was not observed in the control p53-null cells at any time point tested (Fig. 8C, bottom panel).

Finally, the cell death itself does not contribute to a rapid Pol III inhibition by MPA in RAW 264.7 macrophages, as we do not observe its onset within 4 h of MPA treatment, as measured by PARP cleavage and clonogenic survival assays (Fig. 8A and D). In conclusion, our data support no role for p53 in Pol III inhibition upon MPA treatment in mammalian cells.

### Pol III remains partially associated with tDNA templates upon MPA treatment in yeast.

**FIG 9:**
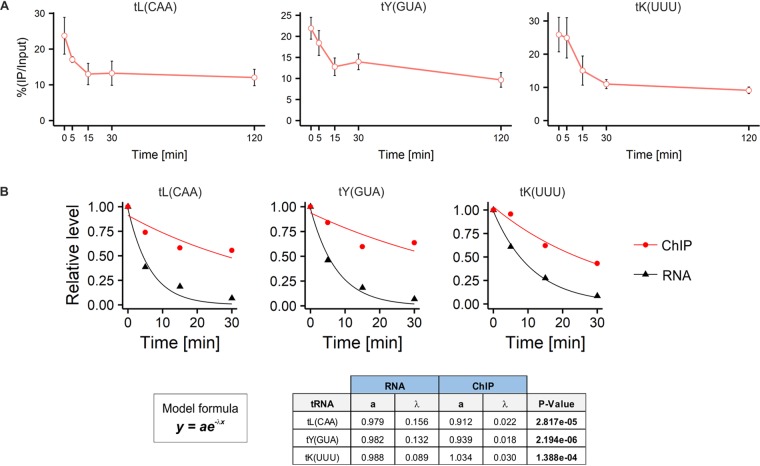
A substantial amount of Pol III complexes is retained on tRNA genes upon MPA treatment of yeast. (A) Wild-type yeast cells expressing HA-tagged C160 subunit were grown in minimal medium without uracil to exponential growth phase at 30°C and then treated with 100 μg/ml MPA for the indicated period. Pol III binding to the indicated tRNA genes was assessed by ChIP assay. Cross-linked chromatin was immunoprecipitated with antibodies to HA followed by RT-qPCR. *n* = 3. (B) (Top) The exponential decay function has been fitted to data from the quantified Northern blots (Fig. 5) and ChIP experiments using the nonlinear least-squares fit method. ANOVA has been performed to test whether there is a significant difference between curves. (Bottom) The model formula and the table with ANOVA results.

To gain a deeper insight into the MPA effect on Pol III transcription, we assessed its impact on Pol III association with tDNA using chromatin immunoprecipitation (ChIP) assays. MPA treatment induces quick but only partial dissociation of yeast Pol III from its template genes. However, the dissociation appeared to be less rapid than the decrease in tRNA levels. Moreover, the substantial portion of Pol III remained associated with tRNA genes after 2 h of MPA treatment (Fig. 9A). The kinetics of Pol III dissociation clearly lag behind the drop in pre-tRNA levels (Fig. 9B). Perhaps, in yeast, MPA treatment, by depleting GTP, prevents transcription elongation without apparent dissociation of Pol III from templates, probably causing its stalling *in vivo*.

**FIG 10:**
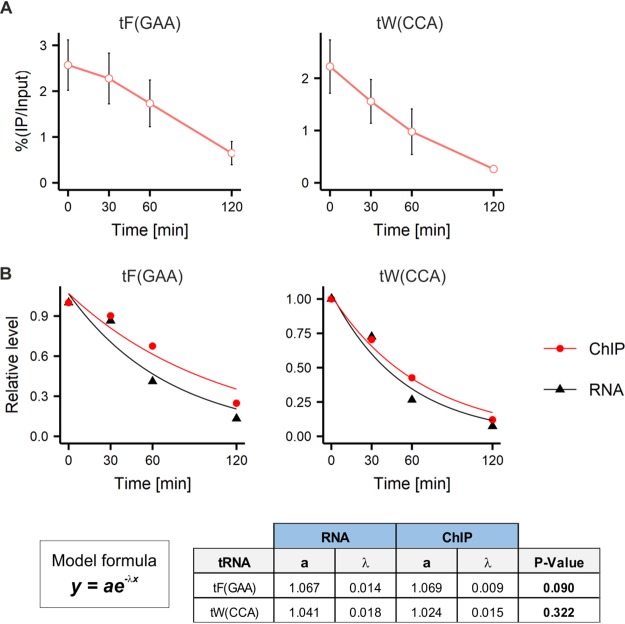
In the RAW 264.7 macrophage cell line, decrease in tRNA levels is accompanied by Pol III dissociation from DNA templates. (A) The RAW 264.7 murine macrophage cell line was left untreated or treated with 10 μM MPA for the indicated period. Pol III binding to the indicated tRNA genes was assessed by ChIP assay. Cross-linked chromatin was immunoprecipitated with antibodies to POLR3D followed by RT-qPCR. *n* = 3. The error bars represent the standard deviation. (B) (Top) The exponential decay function has been fitted to data from RT-qPCR (Fig. 1A) and ChIP experiments using the nonlinear least-squares fit method. ANOVA has been performed to test whether there is a significant difference between curves. (Bottom) The model formula and the table with ANOVA results.

In contrast to yeast, the majority of Pol III complexes in RAW 264.7 macrophages are released from template genes upon MPA treatment (Fig. 10A). Moreover, no significant difference between the kinetics of tRNA transcription inhibition and Pol III dissociation is observed by plotting together the data obtained from RT-qPCR and ChIP assays and fitting the exponential decay function into it (Fig. 10B). Thus, it seems that in a macrophage cell line upon MPA treatment, either Pol III stalling is not occurring or stalled Pol III is rapidly removed by a mechanism that is not present in yeast.

## DISCUSSION

Here, we investigated the effects of MPA treatment on pre-tRNA synthesis in both yeast and mammalian cells, with a special focus on macrophages, an important constituent of the innate immune system. We observed that the cessation of tRNA synthesis by MPA is very rapid in both systems. The decrease in tRNA levels upon MPA treatment in RAW 264.7 macrophages and yeast was clearly rescued upon addition of GMP and guanine, respectively. We excluded Maf1 and p53 as major regulatory contributors to the observed inhibition of Pol III activity upon MPA treatment. Moreover, the downregulation of selected Pol III subunits by MPA seems to be a consequence but not a cause of the decreased Pol III activity. We thus conclude that the direct cause of Pol III inhibition is diminution of guanine nucleotides *per se*. In MPA-treated yeast, Pol III does not dissociate completely from tDNA templates and the substantial fraction of Pol III complexes remains associated with chromatin. These complexes may correspond to DNA-bound, perhaps stalled Pol III transcription units. It is noteworthy that prolonged MPA treatment results in the induction of the *IMD2* mRNA, possibly restoring GTP levels and allowing activation of Pol III complexes associated with tRNA genes (Fig. 2).

Pol III is tightly regulated in response to various growth and stress conditions, and while several different mechanisms are involved in this process, the prominent role is played by the repressor Maf1 (14, 16). Our data, however, clearly showed that in yeast lacking Maf1 and in RAW 264.7 macrophages with knocked-down *MAF1* mRNA, MPA potently inhibits Pol III activity. Similarly, tRNA transcription in HCT116 cells remains sensitive to MPA in the absence of p53, another Pol III repressor. Furthermore, the p53 induction in RAW 264.7 and HCT116 cells occurs much later than the actual cessation of pre-tRNA synthesis. We considered that a decrease in the levels of Pol III subunits may contribute to the response of cells to MPA. We indeed observed downregulation of selected subunits; however, in both yeast and RAW264.7 macrophages, this again occurs later than the pre-tRNA decrease and is therefore likely to be secondary to the decreased Pol III activity. This is consistent with our previously published results showing that in yeast upon metabolic stress, the downregulation of the largest Pol III subunit, C160, is delayed in regard to Pol III activity inhibition (27). We thus concluded that neither Maf1, p53, nor the decrease in Pol III levels is primarily responsible for MPA-induced Pol III inhibition.

In yeast, Pol III dissociates from tRNA genes, but the kinetics clearly lag behind the drop in pre-tRNA levels. Furthermore, under these conditions, Pol III does not dissociate completely even during prolonged treatment. This is strikingly different from the response in yeast cells upon metabolic shift from fermentation to medium with a nonfermentable carbon source, where decreases in tRNA levels and Pol III dissociation kinetics are comparable (27). We speculated that a signaling event(s) triggered by this metabolic shift, which includes Maf1 dephosphorylation (30), actively impinges on Pol III, whereas MPA treatment results in GTP depletion and perhaps Pol III stalling due to the lack of one of the substrates. Pol III may be especially prone to the stalling due to GTP depletion, as it has been shown that incorporation of G by Pol I is slower than that of other residues and that *in vivo* Pol I pauses just before it (31). Our observation is also consistent with the results of *in vitro* transcription studies where a ternary Pol III complex was formed with *SUP4* tDNA as a template in the presence of nucleoside triphosphates (NTPs) without GTP. The SUP4 tRNA does not contain guanine nucleotides within its first 17 nucleotides, which allows for formation of the partial nascent transcript even in the absence of GTP. Importantly, such a ternary complex is stable, and transcription can be resumed upon GTP addition (32). Furthermore, by coimmunoprecipitation experiments performed with extracts of MPA-treated cells, we observed stable interactions between subunits of the remaining undegraded Pol III complexes. These complexes may correspond to the DNA-bound, stalled Pol III transcription units.

**FIG 11:**
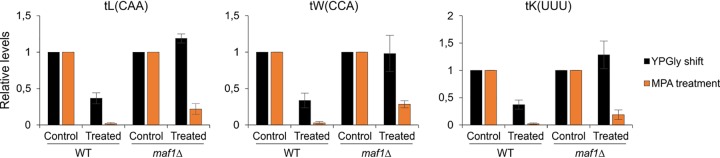
In yeast cells depleted of Maf1, tRNA transcription is regulated differentially in response to metabolic shift and MPA. The levels of indicated tRNAs from wild-type and *maf1Δ* yeast cells nontreated and treated with MPA for 1 h (data taken from Fig. 5) were plotted together with the data from yeast cells shifted to glycerol medium at 37°C for 2 h (data taken from the work of Cieśla et al. [37]). Control samples from both wild-type (WT) and *maf1Δ* yeast cells were set to 1.

Given that MPA efficiently inhibits tRNA transcription in both wild-type and *maf1Δ* yeast cells, it seems that the nucleotide depletion acts independently of Maf1, most likely directly on Pol III. This contrasts with the downregulation of Pol III induced by metabolic stress such as shift to glycerol medium at elevated temperature. In this case, Maf1 is indispensable for repressing Pol III activity, and in the cells depleted of this protein, tRNA downregulation does not occur (Fig. 11). Thus, MPA is the first reported stress-inducing agent that does not require Maf1 to repress Pol III.

## MATERIALS AND METHODS

### Cell culture.

Cells were cultured in a humidified incubator with 5% CO_2_ at 37°C. Murine RAW 264.7, U2OS human osteosarcoma cell line, human colorectal cancer HCT116, and HCT116 p53^−/−^ cells were grown in Dulbecco’s modified Eagle’s medium (DMEM) supplemented with 2 mM l-glutamine, penicillin (100 U/ml), streptomycin (100 U/ml), and 10% fetal bovine serum (FBS), unless otherwise stated. When indicated, cells were treated with 10 μM MPA (Sigma, catalog no. M3536) and rapamycin (BioShop Canada, catalog no. RAP004).

### Yeast culture.

Saccharomyces cerevisiae strains used in this study are listed in Table S1 in the supplemental material. Yeast were grown on synthetic complete medium without uracil (SC−ura [2% glucose, 0.67% yeast nitrogen base containing 20 μg/ml of all the amino acids required for growth except for uracil]). Where indicated, growth medium was supplemented with 50 or 100 μg/ml MPA dissolved in ethanol. To allow growth in the medium without uracil, MW4415 and AC40-GFP C160-hemagglutinin (HA) strains were transformed with an empty plasmid harboring the *URA3* gene, pFL44L (33).

### Transfections.

RAW 264.7 cells were plated on 10-cm dishes at a density of 2.5 × 10^6^. For MAF1 knockdown, cells were transfected with 25 nM small interfering RNA (siRNA) or 4 μg pcDNA Maf1-HA plasmid (34) using Lipofectamine 2000 (Thermo Scientific) according to the manufacturer’s recommendations. The following day, the cells were split into 6-cm dishes. The treatment experiments were performed 2 days after transfections. The siRNAs used were Maf1 (Qiagen, catalog no. SI01307327) and AllStars negative-control siRNA (Qiagen, catalog no. 1027280). U2OS cells were transfected with pcDNA Maf1-HA as described previously (19).

**Protein extracts and Western blotting. (i) Mammalian cells.** Cells were washed with ice-cold phosphate-buffered saline (PBS) and harvested by scraping directly into buffer (100 mM NaCl, 50 mM HEPES [pH 7.9], 1 mM EDTA, 5% glycerol, 0.05% NP-40, 0.1% SDS). Extracts were sonicated in a Bioruptor (Diagenode) and spun for 10 min at 14,000 rpm at 4°C. Supernatants were collected, and protein concentration was assessed using the Bio-Rad protein assay. Proteins were precipitated from a volume of extract corresponding to 100 μg protein with an equal volume of 10% trichloroacetic acid (TCA). Samples were spun for 10 min at 14,000 rpm at 4°C, and the pellet was washed with cold acetone, dried, and resuspended in Laemmli buffer (pH 8.8), followed by incubation for 15 min at 55°C with shaking.

**(ii) Yeast cells.** The protein extraction method was described earlier (35). Twenty micrograms of protein was resolved on an SDS-polyacrylamide gel, transferred to a polyvinylidene difluoride (PVDF) membrane, and incubated with antibodies. The antibodies used are listed in Table S2 in the supplemental material.

### Coimmunoprecipitation.

Coimmunoprecipitation of Pol III subunits with AC40-GFP was performed as described previously (36).

### RNA isolation from mammalian cells and cDNA synthesis.

Total RNA was isolated from cells using TRI reagent (MRC) according to the manufacturer’s instructions. Eighty nanograms of RNA was used for cDNA synthesis using a QuantiTect reverse transcriptase kit (Qiagen). To increase the efficiency of tRNAs’ cDNA synthesis, oligonucleotides specific to the 3′ end of tRNA were added to the reaction mixture, each at the final concentration of 1 μM. The oligonucleotide sequences are listed in Table S3.

### RNA isolation from yeast cells, Northern hybridization, and cDNA synthesis.

RNA isolation and nonradioactive Northern blotting were performed exactly as described previously (27). For Northern hybridization, digoxigenin (DIG)-labeled oligonucleotides were used, and are listed in Table S4. Band intensities from Northern blot images were quantified using MultiGauge v3.0 software (Fujifilm). cDNA synthesis from yeast RNA was performed as described above with the exception that 50 ng of RNA was used.

### ChIP from mammalian cells.

On the day before, 5 × 10^6^ cells were seeded for each time point. Cell cross-linking was done by adding 37% formaldehyde to a final concentration of 1% for 7 min at room temperature, followed by the addition of glycine to a final concentration of 125 mM. Cells were lysed in 1 ml chromatin immunoprecipitation (ChIP) buffer (150 mM NaCl, 5 mM EDTA, 0.5% Triton X-100, 0.5% NP-40, 1 mM sodium butyrate, 50 mM Tris-HCl [pH 8.0]) containing 1.5× cOmplete protease inhibitor cocktail (Roche). The lysate was sonicated 25 times for 15 s with 30-s intervals in a Diagenode Bioruptor set to maximum power. For each IP, 100 μl of extract was used, and 50 μl of extract was used as input. For IPs, extract was diluted five times with ChIP buffer, and 3 μg of POLR3D antibody (A302-296A; Bethyl Laboratories) or, as control, normal IgG rabbit antibody (catalog no. 2729; Cell Signaling Technology) was added. Following overnight incubation at 4°C with rotation, 50 μl of protein G magnetic beads (Dynal Life Technologies) was added, and the mixtures were further incubated for 6 h. Beads were washed twice with ChIP buffer, once with LiCl buffer (250 mM LiCl, 1 mM EDTA, 0.5% sodium deoxycholate, 0.5% NP-40, 10 mM Tris-HCl [pH 8.0]), once with high-salt buffer (500 mM NaCl, 5 mM EDTA, 0.5% Triton X-100, 0.5% NP-40, 50 mM Tris-HCl [pH 8.0]), and one more time with ChIP buffer. All the washes were performed at room temperature for 3 min. Elution of DNA was performed by adding 100 μl of elution buffer (200 mM NaCl, 5 mM EDTA, 0.5% SDS, 25 mM Tris-HCl [pH 7.5]) and incubating for 20 min at 65°C with shaking. Eluates were treated with RNase A (Invitrogen) for 30 min at 37°C and pronase (Roche) for 60 min at 37°C. Chromatin was de-cross-linked by overnight incubation at 65°C. DNA was purified using the QIAquick PCR purification kit (Qiagen) according to the manufacturer’s instructions.

### ChIP from yeast cells.

Yeast ChIP experiments were performed as described previously (37). The input and immunoprecipitated samples were assayed by quantitative PCR to assess the extent of protein occupancy at different genomic regions. Occupancies of Pol III at tRNA genes were calculated by determining the immunoprecipitation efficiency, which is the amount of PCR product in the immunoprecipitated sample divided by the amount of PCR product in the input sample multiplied by 100. Occupancy values were reduced by occupancy on untranscribed fragment of chromosome V (ARS504), which served as a negative control. The PCR primers are listed in Table S5.

### Quantitative PCR.

Quantitative PCR was performed on a Roche LightCycler 480 System using a 3-min incubation at 95°C, followed by 40 cycles of 20 s at 95°C, 30 s at 61°C, and 20 s at 72°C (with a plate read after each cycle). A melting-curve analysis was performed for each sample after PCR amplification to ensure that a single product with the expected melting-curve characteristics was obtained. Each sample was loaded in triplicate. Each plate contained cDNA dilutions for the standard curve, a non-reverse transcriptase control, and a no-template control. PCR efficiencies were between 90% and 100%. Data were processed using LightCycler 480 software and then analyzed in Excel (Microsoft). Data are expressed in arbitrary units calculated from standard curve where the highest cDNA concentration was set to 1. The primer sequences are listed in Table S5.

### Clonogenic assay.

Cells were harvested and counted using a hemocytometer. Cells (5.0 × 10^4^ and 7.5 × 10^4^) were replated on 6-well plates in triplicate for each condition. Next day, the cells were treated with MPA at 10 μM or 0.1% ethanol (as a control) for 4 or 8 h. Then, the medium was removed, cells were rinsed with PBS, and fresh medium without the drug was added. Plates were left in the incubator until cells formed large colonies. Then, medium was removed, and cells were rinsed with PBS, fixed, and stained with a crystal violet solution (0.05% [wt/vol] crystal violet, 1% formaldehyde, 1× PBS, 1% methanol). The cells were subsequently rinsed with PBS, dried at room temperature, and photographed.

### Data modeling.

To compare the kinetics of the decrease in tRNA levels and Pol III dissociation from template genes, first the nonlinear regression method was used to estimate the parameters of the exponential decay function *y = a *×* e*^−λ^*^t^*. This function was chosen as the visual inspection of plotted data suggested that both signals follow exponential decay. To estimate the parameters, a nonlinear least-squares approach was employed using the nls() function in R (38). Then, given the models, analysis of variance (ANOVA) was performed to test whether there is a significant difference between curves.

## Supplementary Material

Supplemental file 1
