# Supplementary material for: Inhibition of tRNA Gene Transcription by the Immunosuppressant Mycophenolic Acid
Source: Mol Cell Biol. 2019 Dec 11;40(1):e00294-19. doi: 10.1128/MCB.00294-19 (PMC6908259; doi:10.1128/MCB.00294-19)
Supplement: Supplemental file 1 [file MCB.00294-19-s0001.pdf]

Jurkiewicz *et al*. – Supplementary data

Supplementary Table 1. Yeast strains used in this study.

Strain	Genotype	Source
MW4415	*MATa ade2-101 lys2-801 leu2-Δ1 his3-Δ200 ura3-52 trp1-Δ63 RPC160::3HA::KanMX6*	(1)
YPH499 C160-HA *maf1Δ*	*MATa ade2-101 lys2-801 leu2-Δ1 his3-Δ200 ura3-52 trp1-Δ63 RPC160::3HA::KanMX6 maf1Δ::URA3*	(2)
AC40-GFP C160-HA	*MATa his3Δ1 leu2Δ0 met15Δ0 ura3Δ0 RPC40::GFP RPC160::3HA::KanMX6*	(3)

Supplementary Table 2. Antibodies used in this study.

Antibody	Secondary antibody	Dilution	Incubation time and temperature	Source
HA	mouse	1:5,000	o/n 4°C	Covance
GFP	mouse	1:2,000	1 h RT	Roche
C82	rabbit	1:10,000	o/n 4°C	A gift from O. Lefebvre
C53	rabbit	1:20,000	o/n 4°C	A gift from O. Lefebvre
AC40	rabbit	1:5,000	o/n 4°C	Custom-made by Gramsh
Pgk1	mouse	1:20,000	1 h RT	Abcam
POLR3A	rabbit	1:2,000	o/n 4°C	1900, (4)
POLR1D	rabbit	1:2,500	o/n 4°C	A304-847A, Bethyl
POLR3D	rabbit	1:2,500	o/n 4°C	A302-296A, Bethyl
PARP	rabbit	1:2,500	o/n 4°C	#9532, Cell Signaling Technology
p53	mouse	1:4,000	o/n 4°C	#2524, Cell Signaling Technology
α-Actin	mouse	1:20,000	10 min RT	MAB1501, Sigma-Aldrich

Supplementary Table 3. tRNA specific oligonucleotides used for cDNA synthesis.

Gene name	Sequence (5' → 3')
Mm tY(GTA)	TCCTTCGAGCC
Mm tW(CCA)	TGACCCCGACG
Mm tF(GAA)	TGCCGAAACCC
Hs tY(GTA)	TCCTTCGAGC
Hs tL(CAA)	TGTCAGAAGTG
Hs tW(CCA)	TGACCCCGACG
Hs tF(GAA)	TGCCGAAACCC

Supplementary Table 4. Oligonucleotide probes used for Northern hybridization (yeast).

**Oligo name**	**Sequence (5' → 3')**
tK(UUU)	ATCCTTGCTTAAGCAAATGCGCT
tY(GUA)	CGAGTCGAACGCCCGAT
tL(CAA)	TATTCCCACAGTTAACTGCGGTCA
tL(UAG)	GCATCCGAAGATATCAGAGATTTAGAGGTTAAATCCA
tW(CCA)	GCAATCTTATTCCGTGGAATTTCCAAG
5.8S rRNA	GCGTTGTTCATCGATGC

Supplementary Table 5. PCR primers used in this study.

**Gene name**	**Forward (5' → 3')**	**Reverse (5' → 3')**
Mm tF(GAA)	TCAGTTGGGAGAGCGTTAGA	TCGAACCAGGGACCTTTAGA
Mm tW (CCA)	AACGGTAGCGCGTCTGACT	ACGTGATTTGAACACGCAAC
Mm tY (GTA)	AGTTGGTAGAGCGGAGGACT	CGAACCAGCGACCTAAGGAT
Mm RPLP0 (ARPP P0)	ACTCTCGCTTTCTGGAGGG	TGACCTTTTCAGTAAGTGGGAAG
Mm ACTB	CCCAGATCATGTTTGAGACC	ATCACAATGCCTGTGGTACG
Mm GAPDH	AAGGGCTCATGACCACAGTC	GGATGACCTTGCCCACAG
Mm 18S rRNA	GCAATTATTCCCCATGAACG	GGCCTCACTAAACCATCCAA
Mm 28S rRNA	TTGAAAATCCGGGGGAGAG	ACATTGTTCCAACATGCCAG
Mm HPRT	AGTCCCAGCGTCGTGATTAG	GGAATAAACACTTTTTCCAAATCC
Mm IMPDH1	ATGGCCTCACCTACAACGAC	AGTGTGATCTTCCGGGTCAG
Mm IMPDH2	TCACTGCAGATCAGGTGGAC	TGACAGTGTCCATGGGTGAG
Mm MAF1	TCTTTAGCTGCCGCTCCATC	CTTCATCAGCCTCCTCTGCC
Hs tY(GTA)	CCTTCGATAGCTCAGCTGGT	CGACCTAAGGATGTCCACAAA
Hs GAPDH	AGGTGAAGGTCGGAGTCAAC	AATGAAGGGGTCATTGATGG
Hs RPLP0	GCGACCTGGAAGTCCAACTA	TGTCTGCTCCCACAATGAAA
Hs ACTB	AAATCTGGCACCACACCTTC	GGGGTGTTGAAGGTCTCAAA
Sc IMD2	TTATGGCTCTGTTGGGTGGT	ACCAACGGTCGTAGTTGGAG
Sc ACT1	CATGTTCCCAGGTATTGCCGA	GTCAAAGAAGCCAAGATAGA
Sc ALG9	TCCATGATACAGGAGCAAGC	CTACCATCAGAACCGCATTC
Sc TDH	GGTATGGCTTTCAGAGTCCCA	AGACAACGGCATCTTCGGTG
Sc tL(CAA)G1	AGAACCGAAACATACAAATAAG TGGT	TGATCACAGAACCAAAAAGATAAAA
Sc tY(GUA)J1	TAATCATTTATCACTCTCGGTAG CC	GGCCAAGAGAAATTTCATCCAC
Sc tK(UUU)P	ACAACACGCTCCGAAGAACT	CAATATGTATTGTCTTTGTTTCTGCAT
Sc ARS504 (control region)	CTGTCAGAATATGGGGCCGTAG	CCATACCCTCGGGTCAAACAC

1. Soutourina J, Bordas-Le Floch V, Gendrel G, Flores A, Ducrot C, Dumay-Odelot H, Soularue P, Navarro F, Cairns BR, Lefebvre O, Werner M. 2006. Rsc4 connects the chromatin remodeler RSC to RNA polymerases. Mol Cell Biol 26:4920-33.
2. Ciesla M, Skowronek E, Boguta M. 2018. Function of TFIIIC, RNA polymerase III initiation factor, in activation and repression of tRNA gene transcription. Nucleic Acids Res 46:9444-9455.
3. Lesniewska E, Ciesla M, Boguta M. 2019. Repression of yeast RNA polymerase III by stress leads to ubiquitylation and proteasomal degradation of its largest subunit, C160. Biochim Biophys Acta Gene Regul Mech 1862:25-34.
4. Fairley JA, Scott PH, White RJ. 2003. TFIIIB is phosphorylated, disrupted and selectively released from tRNA promoters during mitosis in vivo. The EMBO journal 22:5841-50.
